# Dystrophin rescue in the brain for DMD

**DOI:** 10.1016/j.omtn.2025.102827

**Published:** 2026-01-14

**Authors:** Mengzhen Li, Renzhi Han

**Affiliations:** 1 Department of Pediatrics, Herman B Wells Center for Pediatric Research, Indiana University School of Medicine, Indianapolis, IN 46202, USA

## Main text

Duchenne muscular dystrophy (DMD) has long been characterized by progressive skeletal muscle degeneration^1^; however, increasing evidence shows that loss of dystrophin isoforms in the central nervous system (CNS) significantly contributes to cognitive, emotional, and behavioral abnormalities.^2^ Several isoforms of dystrophin such as Dp427, Dp140, and Dp71/Dp40 are expressed in brain, playing important roles in neuronal stability, synaptic signaling, and higher-order cognitive processing. Their absence correlates with heightened anxiety, impaired learning, attention deficits, and autism-spectrum-related traits.^2^ Although major therapeutic advances have been made in muscle-directed gene therapy for DMD, including Food and Drug Administration (FDA)-approved micro-dystrophin gene therapy delivered using adeno-associated virus (AAV) and antisense oligonucleotide (ASO)-based exon-skipping treatments,^3^ dystrophin deficiency in CNS remains unaddressed and represents a critical gap in current therapeutic strategies.

**Figure 1:**
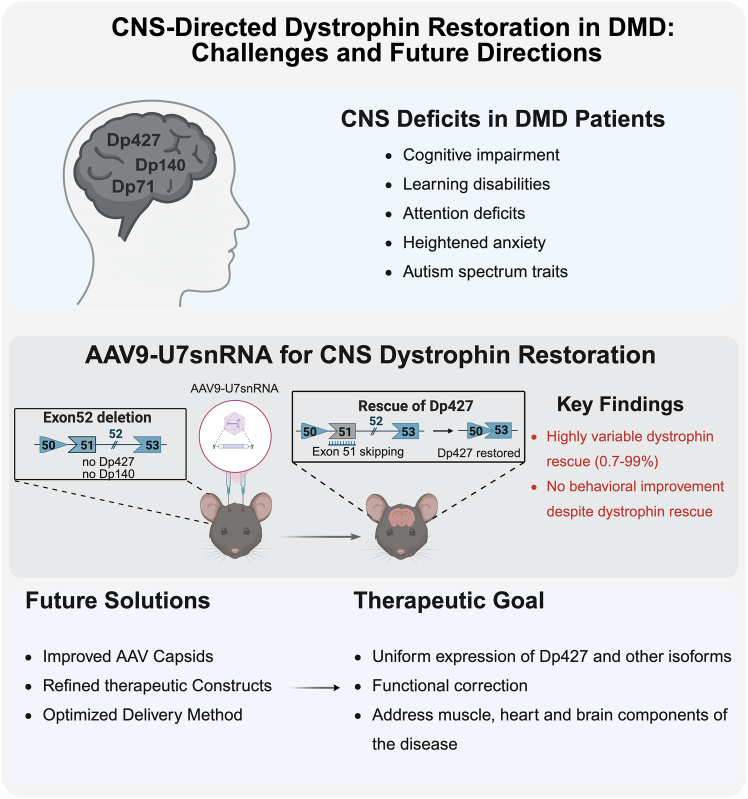
CNS-directed dystrophin restoration in DMD

A recent study by *Vacca* et al. published in *Molecular Therapy* *-* *Nucleic Acids* investigated whether AAV9-delivered U7snRNA-mediated exon 51 skipping could restore truncated Dp427 expression in the brain of mdx52 mice and improve the associated neurobehavioral deficits (Figure 1).^4^ Previous work from the same group showed that the mdx52 mouse model, which lacks Dp427, Dp260, and Dp140 in the brain, displays pronounced anxiety-related phenotypes and defective fear learning.^5^ They recently demonstrated that a single intracerebroventricular (ICV) injection of tricyclo-DNA ASO targeting exon 51 restored 5%–15% of Dp427 expression and led to reduced anxiety and unconditioned fear.^6^ Another study using PMO-ASO-induced exon 53 skipping restored Dp140 expression and improved the abnormal social behavior in the same mouse model.^7^ These studies suggest CNS abnormalities in DMD could potentially be reversed, at least partially by dystrophin rescue, raising hope for future brain-targeted gene therapies.

In this study, the authors evaluated whether a vectorized AAV9-U7snRNA approach could enhance the magnitude and durability of CNS dystrophin restoration. Using bilateral ICV delivery of scAAV9-U7-EX51M at both low (2E11 vg) and high (2E12 vg) doses, the authors assessed dystrophin restoration in mdx52 mice 7 weeks after treatment. Despite successful induction of exon 51 skipping, AAV9-U7snRNA treatment resulted in highly variable dystrophin rescue. Dp427 RNA restoration ranged from 0.7% to 99%, and protein recovery exhibited similarly broad heterogeneity. Notably, the authors stated that this degree of variability had not appeared in their previous ASO studies using the same bilateral ICV procedure, indicating that the inconsistency likely stems from inherent biological limitations of the AAV9-U7snRNA approach, such as uneven vector distribution, cell-type-dependent transduction, and variable U7snRNA expression, rather than simply from technical factors.

Despite detectable dystrophin rescue in different brain regions involved in emotional regulation and learning, treated mdx52 mice exhibited no significant changes in anxiety-like behavior or fear-learning performance. This contrasts with their previous ASO studies in mdx52 mice, where above 4% of Dp427 rescue across all three analyzed brain regions was found to show measurable functional improvement. These findings highlight a key therapeutic consideration: effective CNS therapy likely requires spatially uniform dystrophin rescue at substantial levels. Low levels or region-restricted restoration would unlikely correct complex neural circuitry deficits.

These challenges reflect broader obstacles in CNS-directed gene therapy. While AAV-based strategies have shown robust efficacy in skeletal and cardiac muscle,^8^^,^^9^ the CNS imposes distinct barriers, including limited vector dispersion, heterogeneous cellular populations, and higher expression thresholds required for behavioral correction. Achieving meaningful dystrophin restoration in the brain will likely require improved delivery platforms capable of far more uniform vector distribution and precise control of transgene expression. Encouragingly, recent breakthroughs in neurotropic AAV development are directly confronting these limitations. In particular, advances in capsid engineering have generated vectors such as BI-hTFR1,^10^ which exploits the human transferrin receptor for enhanced CNS targeting, holding tremendous translational potential compared to variants derived from directed evolution in mice, which often results in capsids with limited cross-species CNS tropism. These next-generation vectors markedly improve systemic CNS delivery by boosting blood-brain barrier penetration and enhancing tropism for defined neural cell populations. Complementary to systemic approaches, the use of intrathecal (IT) or ICV administration methods remains a vital approach, often synergizing with these newer capsid designs to maximize local concentration while potentially mitigating the overall systemic load.

Beyond improved biodistribution, these engineered capsids also provide a foundation for more sophisticated therapeutic strategies. Their enhanced efficiency creates opportunities to deliver either oversized full-length therapeutic genes^8^ or optimized truncated constructs, thereby addressing both expression thresholds and AAV packaging constraints. At the same time, the ability to combine these vectors with cell-type-specific promoters, regulatory elements, and refined dosing paradigms may further improve spatial precision and safety factors that are particularly critical in the CNS. Importantly, the translational potential of these approaches will depend on careful evaluation of species-specific tropism, long-term expression stability, and dose-related safety considerations. Future CNS-directed strategies for DMD will also need to integrate advances in capsid engineering with equally sophisticated genetic designs that account for the complexity of neural circuits and developmental context. As such, next-generation AAV capsids, when combined with optimized gene constructs and delivery paradigms, may substantially advance the development of effective CNS-targeted therapeutic strategies for DMD.

In summary, the work provides important insights into both the promise and the limitations of AAV9-U7-mediated exon skipping for CNS dystrophin restoration. Its findings reinforce the need for next-generation CNS delivery strategies, whether through improved capsid design, enhanced splicing tools, or alternative gene modulation platforms, to achieve uniform, functionally meaningful levels of dystrophin expression. Addressing these challenges will be essential for developing comprehensive therapeutic solutions for DMD that target both the muscle pathology and the neurological components of the disease.

## Acknowledgments

R.H. is supported by US National Institutes of Health grants (R01HL170260, R01HL169976, and R21HL163720).

## Declaration of interests

R.H. is a Section Editor of *Molecular Therapy,* an editorial board member of *Molecular Therapy – Nucleic Acids*, and a founder of Zhida Therapeutics with interests in developing gene therapies.
